# Partnership of I-ACT for children (US) and European pediatric clinical trial networks to facilitate pediatric clinical trials

**DOI:** 10.3389/fped.2024.1388170

**Published:** 2024-11-07

**Authors:** Eva Degraeuwe, Collin Hovinga, Annelies De Maré, Ricardo M. Fernandes, Callie Heaton, Lieve Nuytinck, Laura Persijn, Ann Raes, Johan Vande Walle, Mark A. Turner

**Affiliations:** ^1^ Department of Internal Medicine and Pediatrics, Ghent University, Ghent, Belgium; ^2^ Critical Path Institute, Tucson, AZ, United States; ^3^ The Department of Pediatrics, University Hospital of Ghent, ERKNET Centre, Ghent, Belgium; ^4^ conect4children Stichting, Utrecht, Netherlands; ^5^ Laboratory of Clinical Pharmacology and Therapeutics, Faculty of Medicine, University of Lisbon, Lisbon, Portugal; ^6^ Institute for Advanced Clinical Trials for Children, Rockville, MD, United States; ^7^ Institute of Life Course and Medical Sciences, University of Liverpool, Liverpool, United Kingdom

**Keywords:** pediatric, drug development, networks, metrics, global

## Abstract

**Background/aims:**

Due to a lack of standard pediatric prescribing information, medicines are often used in a dosage form or for an indication that has not been investigated in children. Pediatric clinical trial research networks aim to facilitate the timely availability of innovative drugs for children by developing standardized trial facilitation and conduct processes. This paper aims to assess the (pre)feasibility duration and characteristics of a US-sponsored clinical trial, in collaboration with I-ACT for Children, for distribution across European sites via European clinical research facilitation networks.

**Method:**

A transatlantic partnership between the Belgian Pediatric Clinical Research Network (BPCRN,) and I-ACT for Children conducted feasibilities in Europe for industry-sponsored early-stage pharmacological clinical trials between 2019 and 2022. The collaboration recorded time to event for key elements of feasibility, influences on successful feasibility, and benefits of collaboration.

**Results:**

Trials were conducted across 17 European countries with 202 participating hospital sites. The initial phase, the pre-feasibility questionnaire had a 70% response rate from 142 sites, and sites took a median 38 days (IQR 20 days) to complete the questionnaire for five trials. All responses underwent a quality control, addressing inaccuracies in site capabilities and recruitment. The first trial's CDA and feasibility questionnaire were completed in roughly 2 months for 7 countries. Time to completion was affected by precontracted sites, limited scope of studies, changes in timelines, COVID-related disruptions, and a learning curve for collaboration.

**Conclusion:**

Collaboration between European collaborative national networks and US-network I-ACT for Children has supported site identification of global pediatric clinical trials. This illustrates one method for the importance of early engagement with sponsors and implementation of effective communication systems.

## 1 Introduction

Globally, due to a lack of standard pediatric prescribing information, clinicians often use medicines in a dosage form or for an indication that has not been adequately investigated in children (1). During the past 20 years, regulators have acknowledged the importance of studying drug safety and efficacy in a pediatric population through the Best Pharmaceuticals for Children (BCPA, US), the Pediatric Equity Act (PREA, US) and the EU Pediatric Regulation in 2007 (2–4).These legislations have had notable successes, resulting in more than 700 changes in US Food and Administration (FDA) product labels as well as doubling the amount of pediatric clinical trials being conducted in Europe (5–7). Notwithstanding these successes, many development-driven pediatric clinical trials in the last decade failed to achieve their intended goals on time (3). Although there are several factors that contributed to this outcome, a significant reason was the inability to recruit a sufficient number of patients within the required time frame to achieve meaningful results to require labelling (8, 9). Since the need for pediatric trials will be increasing further, especially in the case of pediatric rare diseases, the challenges will only become greater (10, 11).

To address the challenges inherent in conducting pediatric clinical trials, international networks can play a pivotal role by establishing and implementing a standardized process to support trial conduct through an overarching central network point and operations of a network on national or site level. This standardized approach would enhance efficiency by reducing administrative burdens and expediting trial timelines. Furthermore, these networks have the potential to optimize study design by providing input that sets realistic expectations for site and patient recruitment and ensures more successful and meaningful outcomes in pediatric clinical research. Networks can cover scattered geographical areas and group knowledge of jurisdictional as well as practical barriers (12–15). Overcoming these barriers is key to global interoperability focusing on intent-to-label trials and ultimately accessibility to adequate drugs for all children (16).

An established disease agnostic pediatric clinical trial research network is the Network for Advanced Clinical Trials (I-ACT) for Children, founded in 2017 in the US. The network was established through a grant from the FDA, memberships by biopharmaceutical companies, and philanthropy. The goal of the network is to facilitate the timely availability of innovative drugs for children, through a network of sites as well as expert facilitation regarding innovative methodologies in pediatrics. Examples include Bayesian statistics, adaptive trial designs, master protocols, decentralized trials, amongst others (14).

In response to gaps identified in the 5 and 10 year reviews of the EMA regulation, a multi-country pediatric clinical trial research network was setup with support from the Innovative Medicines Initiative (IMI2) public-private consortium, conect4children (c4c, www.conect4children.org) founded in 2018 in Europe (17, 18). The research network is focused on four main areas of services: strategic feasibility expert advice, an academy for education and training, support for managing data, as well as a network of over 200 sites following harmonized procedures coordinated through 20 National Hubs hosted by pediatric clinical research national networks and a central Network Infrastructure Office. The time limitation for the consortium extends until 2025, encompassing the 21 European beneficiaries and the 10 EFPIA partners involved in activities outlined within the grant agreement (No 777389). Engagement of c4c with other international networks has been ongoing from its initial stages, but operational collaboration is limited during the project (17, 18). A new legal entity, the conect4children Stichting has been established to ensure sustainability of this project's results, services and activities. A Mordor Intelligence Report, published in 2023, state that US continues to have the largest pediatric clinical output globally, followed by Europe. Both regions’ output is expected to further increase, with a total compound annual growth rate of 14.5% in the coming five years (17).

Pediatric clinical trials must adhere to regulatory requirements, which differ between jurisdictions. Conducting separate trials in each continent is expensive and not always feasible. Connecting pediatric clinical trial networks across continents could improve efficiency by sharing resources and data while meeting quality standards, ensuring consistency and reliability of trial results. To explore the value of collaborating across multiple jurisdictions, a partnership was formed between I-ACT for Children, based in the United States, and European pediatric clinical research national networks that also collaborated in the conec4children project. The Belgian Pediatric Clinical Research Network (BPCRN) served as a contact person transatlantically with I-ACT for Children from 2018 to 2022, as an intermediary with the 20 other national collaborative networks as a proof-of-concept. This collaboration is unique in that it represents the first transatlantic partnership focused on clinical trials for both common and rare pediatric diseases.

This paper has three aims:

1. To provide an overview of the partnership between I-ACT for Children and the European pediatric national networks in c4c mediated by BPCRN.
2. To describe the partnership's experience of conducting early engagement surveys (EES) and site feasibility questionnaires (FQ) for pediatric clinical trials sponsored by industry
3. To provide a case study of collaboration between c4c and I-ACT for Children.

## 2 Methods

### 2.1 Partnership I-ACT for children and the European research networks

I-ACT for Children is a non-profit organization based in Maryland, USA, that operates as a neutral and independent entity. The organization has established site networks across multiple regions, including Australia, Latin America, Saudi Arabia, and a Canadian Network affiliation.

The Belgian Clinical Trial Network within the Belgian Pediatric Clinical Research Network the BPRCN, is based in Ghent, Belgium.

The conect4children (c4c) consortium was under the IMI2 grant restricted to conduct proof-of-viability studies from a handful of selected EFPIA partners and limited to 7 trials over these partners until April 2024. The sponsors that I-ACT is hired for, are mostly US-based industry partners that did not submit a trial request within the grant. Therefore, BPCRN, one of c4c connected networks, had to function as a contact person between I-ACT for Children and the other c4c-connected networks to facilitate pediatric clinical trials in Europe. These other c4c-connected networks include is the collaborative pediatric national networks of Italy (INCIPIT), The Netherlands (PEDMED-NL), Switzerland (SwissPedNet), Portugal (Stand4Kids), Estonia (ELAV), Austria (OKIDS), Belgium [Belgian Pediatric Clinical Research Network (BPCRN)], Greece (HELPNET), Poland (POLPEDNET), Norway (NORPEDMED), Germany (GermanNetPaet), Ireland (IN4KIDS), Sweden (SWEDPEDMED), Spain (RECLIP), Hungary (MCRN-Hungary), Finland (FINPEDMED), France (PEDSTART), Czech Republic (CzechPharmNet), Denmark (DANPEDMED), and has knowledge of submission for the UK. All the involved collaborative networks have been involved within Networking for National Networks (NNN) that could be used to communicate for I-ACT for Children studies conducted in Europe. More information and corresponding geographical figure of the collaborative pediatric national networks can be found on https://conect4children.org/national-hubs/.

All c4c networks have activities other than trial facilitations of intent-to-label trials, such as experts’ advice and trainings, which will not be included within the scope of this paper. Both European Research Networks as well as I-ACT for Children focus on human studies within neonates, children and adolescents.

The Partnership's formation and development are described qualitatively. The nature and the content of each network, and their joint work were discussed during regular meetings with the coordinating stakeholders (BPCRN, I-ACT for Children, respective industry sponsors) during 2020–2022. To foster alignment of the I-ACT for Children and European pediatric clinical research national networks, an initial consultation was organized to familiarize both networks with leadership and operationally involved staff. This process included conducting virtual meetings with representatives from both networks to pinpoint their main challenges and needs. After these discussions, there was a phase of gathering data from the meetings, which led to the creation of collaboration conduct standardization as well as five trial projects to test the partnerships.

The five trials are from US-based sponsors that I-ACT for Children has been contracted to facilitate site identification and post-side identification optimalisation (site communications or recruitment optimalisations). All trials were rare disease indications. This study focuses on the metrics of the European facilitation by BPCRN together with the other European research networks, and does not include the parallel US-based facilitation performed by I-ACT for Children. However, we must note that US facilitation had the same milestones for completion as the European facilitation, and therefore follow a similar trend. Additionally, the questionnaires used in the five studies had a varying length, where early engagement or pre-feasibilities entailed 8–10 questions and feasibility questionnaires (requiring full protocol access) 10–24 questions. Both types of questionnaires were used for sponsors to be able to select sites for their trial. The focus of trial facilitation being on the pre-feasibility questionnaire without confidentiality disclosure agreement (CDA) and/or feasibility questionnaire (FQ) with CDA was at the liberty of the sponsor, which is a multifactorial decision (budget, experience with trial facilitation networks, initial outreach and timelines, complexity of the trial). The services of a trial facilitation network can also include input on how to improve site communication and recruitment in a third phase, shown qualitatively in Table 1 and quantitatively in Table 2.

**Table 1:** Services offered in the transatlantic partnership between BPCRN and I-ACT for children to industry sponsors, including the activities and roles of the network.

Stage of the clinical trial	Activity	Role of the network
Early Outreach	- Identifying countries with capable sites in the therapeutic domain and estimating the number of sites at the national level.	- Evaluating site accuracy and capability in real-time by country managers.
Early Engagement Survey (EES)	- Collecting preliminary information from site champions or established single points of contact - Examples include recruitment estimates, non-confidential protocol requirements, timelines, and direct contact information.	- Reviewing necessary questions and adjusting for national differences, facilitating troubleshooting directly in the national language. - When responses received, the responses are checked for accuracy and completed when missing data. - A comparison is made nationally or globally for realistic representation and a summary of the findings with advantages and disadvantages per site is provided.
Confidential Disclosure Agreement	- Securing confidentiality before sharing the protocol, either through a master-agreement or the template of each industry partner.	- Performing a first review of the agreement, adjusting to necessary national or site-level changes, and sending it to accurate and updated contacts at site level. - Performing a final review of accuracy before sending to the sponsor’s contracting services.
Feasibility Questionnaire (FQ)	- Completing a lengthier questionnaire detailing infrastructure requirements, submission and start-up timelines, screening rates and failures, and multidisciplinary review sections.	- Reviewing of the questionnaire (for National adaptations) before dissemination. - Making detailed summary reports per site as well as recommendation of improvement points during the site initiation visit (SIV). - Sharing information directly to the sponsor, in either report style or full presentation, aside from in real-time updates of the trial progression.
Handover of sites to the Clinical Research Manager of the Sponsor	- Selecting, backing up, or declining a site, and handing it over to the sponsor’s clinical research manager for validation and preparation for site opening.	- The network shares the information directly and provides a smooth handover so that each site is faced with only one trial study point of contact per phase.
Trial Start-up and Facilitations	- Overcoming secondary hurdles, such as unresponsiveness, submission delays, and recruitment difficulties during trial commencement.	- Supporting trial progress as a standby facilitator to both the site and sponsor due to personal peer-to-peer connections with the site.
Trial Communications	- Evaluating trial progress and providing updates to the global sponsor team, I-ACT for Children project manager, and respective subcontractor network leads.	- Sharing updates in real-time according to the sponsor’s preferences, with a maximum of one working day delay.

**Table 2:** Metrics of 5 facilitated industry-sponsored trials through the European collaborative networks within Europe.

Trial	Therapeutic area	Timeframe	Number of countries involved within Europe	Number of Early Outreach European sites	EES completion (sites/%)^a^	EES completion in days [median, (IQR)]	CDA	FQ	Feasibility completion in days	Example of Trial Facilitation areas after feasibility
1	Neurology	May to September 2020	7	42	**29** (**69%)**	30	**25**	**25**	**54**	Trial start-up and site communications.
2	Dermatology	March to May 2021	7	30	**25** (**83%)**	35	/	/	/	UK-platform submission and national/site comparison upon collection.
3	Infectiology	July to August 2022	4	64	**40** (**63%)**	56	/	/	/	Approaching sites outside of the established network.
4	Cardiology	March to November 2022	12	56	**42** (**75%)**	42	/	/	/	Revisiting declined sites by the sponsor.
5	Neurology	August 2022	2	10	**6 (60%-**	28	/	/	/	Investigating duration of conflicting trial per site.
All			**32**	**202**	**142** (**70%)**	38 (IQR 20)				

EES, early engagement survey; CDA, confidentiality disclosure agreement; FQ, feasibility questionnaire.

^a^ including data cleaning, approaching incomplete surveys and summarizing findings.

Bold is the EES timelines, the most relevant metric of the table.

Structured sponsor discussions, combined with insights from I-ACT for Children and European network feedback, have outlined trial stages, activities, and network roles. The multistakeholder feedback was collected during 2020–2022 and summarized according to relevance by both networks for a wider audience within Figure 2 and Table 3.

**Table 3:** Case studies on trial facilitation: impact on site outreach and Non-standard services provided.

Facilitation	Applicable trials	Description
Early Outreach	Trial 1–5	- Sponsors plan to approach countries with previous trial experience, preferably within the indication. - However, sponsor experience is often limited to adult physicians and clinical trial units. - A network’s input on the most interesting **countries** and **site estimation** is crucial for strategically planning paediatric clinical trials.
EES	Trial 2	- **Peer-to-peer support** for low-experienced Principal Investigators (PIs). - A low-experienced PI at a smaller site was contacted in their local language to investigate a situation due to unresponsiveness to the EES. - The interpretation and barriers for completing the EES were resolved, and the site’s odds were improved for inclusion in the trial.
EES	Trial 3–4	- **Pandemic regulations and fluctuations** influenced the site’s capabilities to perform a trial. - A wave-model was constructed where trial outreach for different countries was compartmentalised within trial timelines through different outreach waves. - Sites received support to reorganize the clinical trial unit to continue performing trials.
EES	Trial 4	- Collaborative networks investigated the concept of **decentralized** trials to reach site opening, especially in low population density countries and/or pandemic situations.
EES	Trial 3–5	- Some sites declined due to **limited time or misinterpretation** of the non-confidential summary and/or wording of the questionnaire. - **Personal connections** with sites allowed revisiting responses, resulting in a 28% increase in **inclusion** of earlier sites that declined the trial request for one trial.
EES (& FQ)	Trial 1–5	- Question-response evaluation found that over **half of the completed questionnaires required adaptations** (one or multiple questions, missing data, unrealistic responses, amongst others) in interpretation by the PI/site team, which could be compared to similarly capable sites over different countries. - Majority of the **corrections** were based in site availabilities, recruitment estimates, and relevant experience, creating more realistic expectations for both site and sponsor. - **Summary reports** overviewed the advantages, disadvantages, strategic advice, and continent-wide evaluation of each trial recruitment goal in correspondence with the sponsor.
Other Trial Facilitations	Trial 1, 3, 4	- **Unresponsive site**: having contact details of multiple PIs and multidisciplinary contacts, a network was able to reactivate a site and plan a site initiation visit in a site where previous contact was troubled.
Other Trial Facilitations	Trial 2	- **Other paediatric disciplines**, such as Dermatology, which are usually not physically located within the paediatric departments, required an extension of contacts and knowledge of the interaction with the CTU per site.
Other Trial Facilitations	Trial 4	- Per discipline national coordinators or in some cases discipline **committees evaluated the trial request** upon early outreach for availability per country.
Other Trial Facilitations	Trial 1, 3, 4, 5	- **Communication clarifications** were needed when PIs were contacted through **multiple routes** (sponsor, networks, CRO’s) causing confusion and hesitancy by the PI to participate. - A peer-to-peer personal phone call improved communication and reached a conclusion within one working day.

EES, early engagement survey; PI, principal investigator; FQ, feasibility questionnaire; CTU, clinical trial unit.

### 2.2 Experience with site identification

The experience of site identification is described both quantitively and qualitatively. A quantitively study was conducted on five pediatric industry trials that were supported by European pediatric collaborative clinical trial networks as subcontractors of I-ACT for Children, with the Belgian Network leading the effort from 2019 to 2022. The process of identifying the site was initiated based on the results of the EES and the completion of the FQ This step in identifying the site was part of the milestone-driven approach adopted by I-ACT for Children.

The process of tracking internal time was organized around two specific milestones. The data, which quantifies the duration of these milestones, is expressed in calendar days to facilitate comparison with other research networks. This data includes median and interquartile ranges (IQR) where more than 1 type of the trial was conducted. The first milestone is initial site outreach and conducting pre-feasibilities or EES. The second milestone is the conduct of CDA, FQ and where requested a summary per site. This milestone's duration was measured during a specific trial and is presented as the number of days required for completion. Since the CDA completion times were not tracked separately for each phase, the report provides a range of days spanning from the earliest CDA completion to the latest. Additionally, the report includes a total count of sites involved.

For countries participating in more than one trial, a correction was made to remove duplicate entries, ensuring that only unique countries are included in the results. However, the analysis does not cover a comparison between master CDAs and CDAs based on industry templates.

Both surveys (EES and FQ) were collected through REDCap (version 10 or higher, Vanderbilt University, Tennessee, USA). Quality control review was conducted within REDCap or MS Excel (version 2020 or higher). Quality summary reports were constructed in Microsoft Word (Microsoft office 2021) through a template developed by I-ACT for Children. Time estimations of summary reports were not standardly collected and were estimated based on calendar logging of the tasks.

The qualitative analysis examined internal communications to identify key differences and obstacles encountered across different continents. The selection of these data points was based on their relevance to the study's objectives and the feedback from both networks in regard to the trial facilitation [including the (pre)feasibility services and post site selection services]. Subsequently, detailed descriptions were formulated, which captured the essence of each facilitation activity and its outcomes in the trials. These descriptions were derived from direct observations, internal communications with sponsor, site and the research networks, and post-milestone feedback loops within the trial process. The descriptions were captured after every meeting and collected in the Tables 1, 3. Each entry in the Tables 1–3 was then cross-referenced with the primary data sources to ensure accuracy and completeness. The finalized tables provided a structured overview, categorized per facilitation types with applicable trials and their corresponding descriptions. The data was captured for Europe alone.

## 3 Results

### 3.1 The partnership

**Figure 1:**
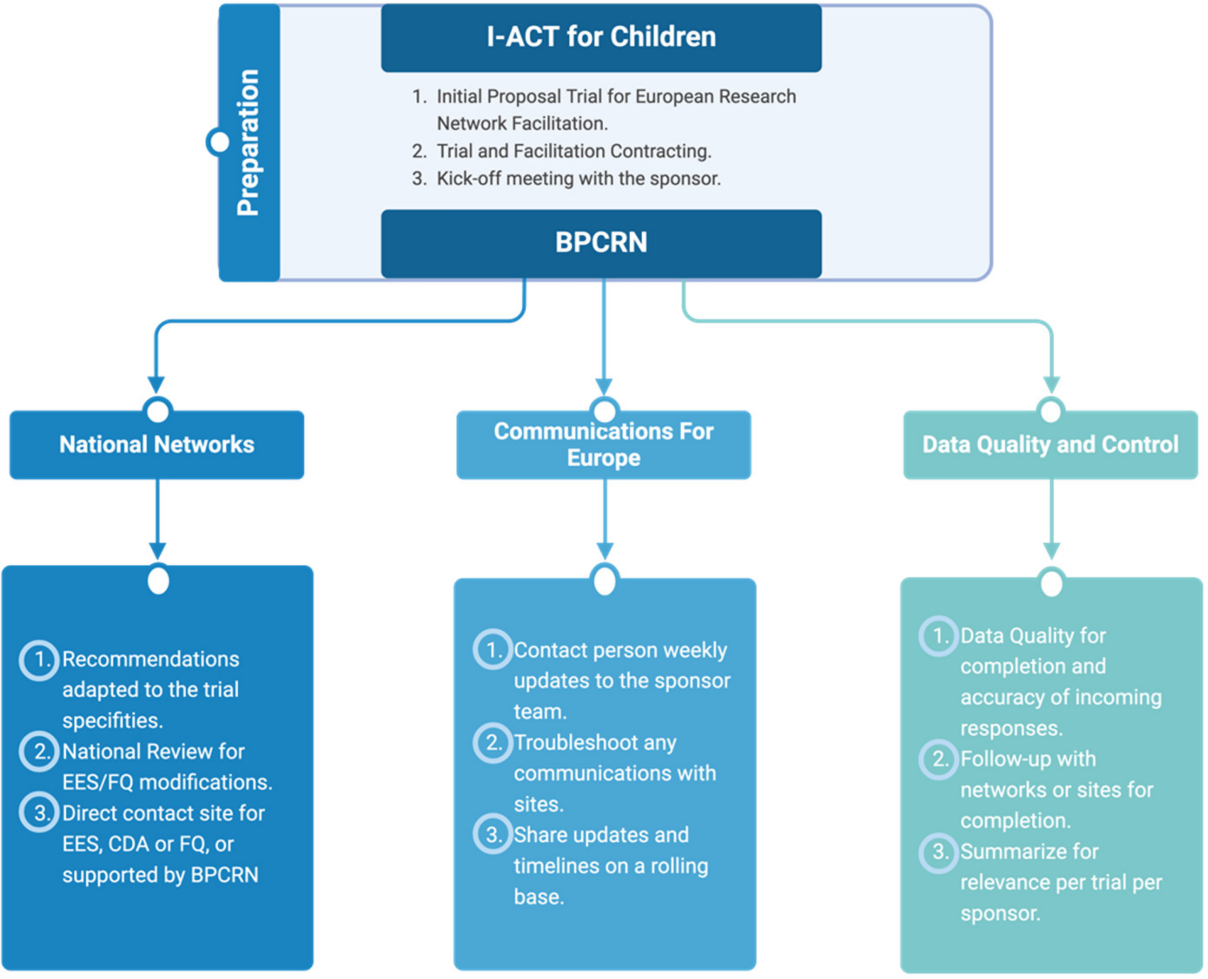
The standardization of the collaboration, and the practical conduct of the trial facilitation. EES, Early Engagement Survey; CDA, confidentiality disclosure agreement; FQ, feasibility Questionnaire; BPCRN, Belgian Pediatric Clinical Research Network. Created by Biorender (2024).

In 2019, the first steps were undertaken to standardize the collaboration. In agreement with c4c, the Belgian Pediatric Clinical Research Network was approved as the liaison between I-ACT for Children and the collaborative pediatric national networks. A confidential disclosure agreement was executed between both networks. The standardization of the collaboration, and the practical conduct of the trial facilitation is shown in Figure 1.

The activities of the network Partnership are summarized in Table 1.

**Figure 2:**
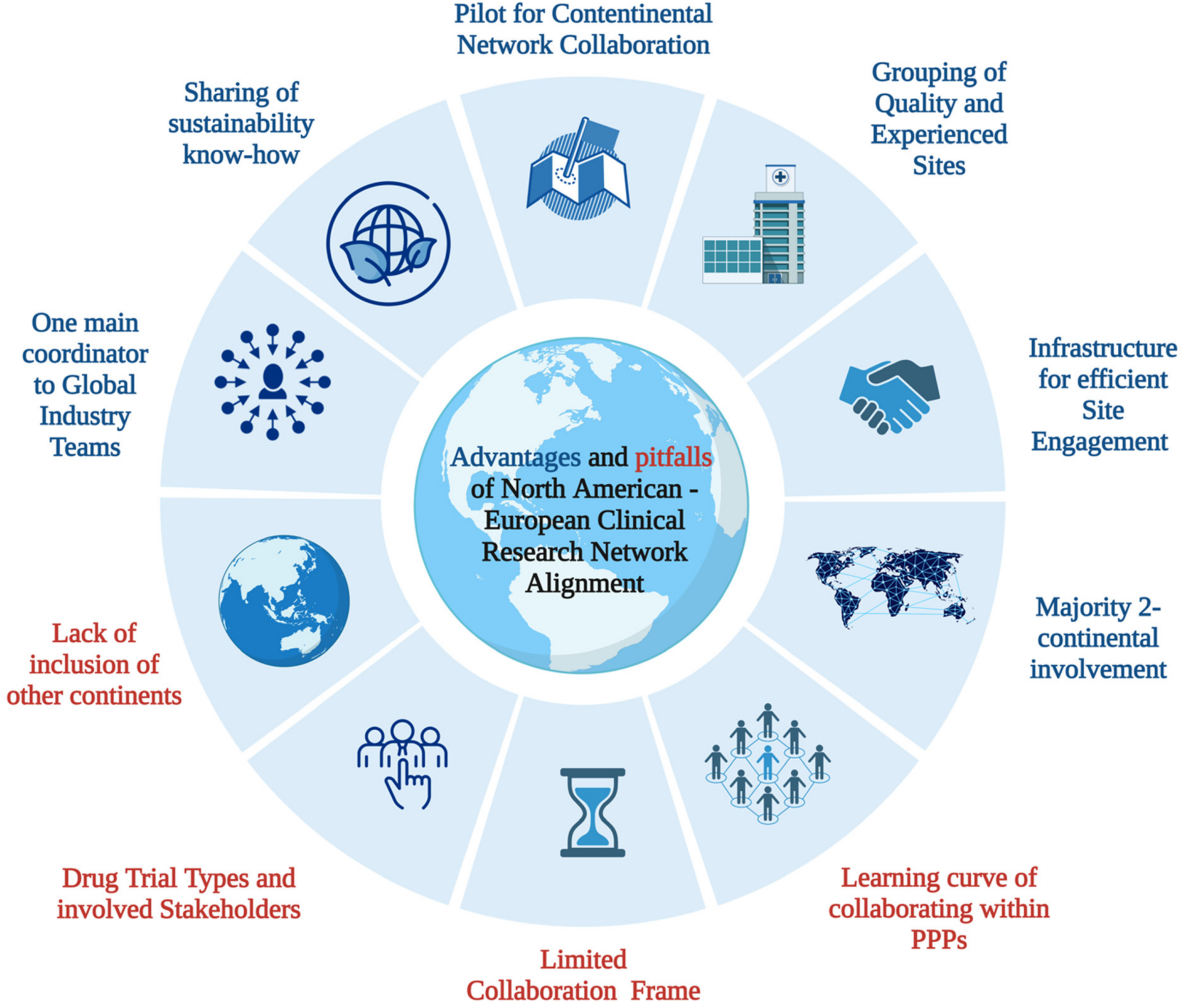
Overview of preliminary analysis: advantages and pitfalls of North America—European paediatric clinical research network alignment. CRO, clinical research organisation; PPP, public-private partnership. Based on template “Risk Factors of colorectal cancer’, created by Biorender (2023).

Figure 2 summarizes the analysis of both the advantages and pitfalls of collaborating at the start of the partnership. The analysis revealed that one main advantage of collaboration is having a central point of contact for the global industry team, which can utilize the established infrastructure and expertise in each country. Consequently, the central point of contact of each respective network can share local knowledge and strategic advice with ease during the early stages of the outreach process. Pilot partnership enhance international collaboration, coupled with insights from PPP collaborations. Nevertheless, the analysis also identified some main pitfalls, including the three-year trial performance timeframe and the need to incorporate the learning curve of the collaboration within this period.

I-ACT for Children's infrastructure includes master CDA with their sites, which expedite the process for FQ requests. Since the master CDAs are pre-executed, they allow immediate progress to FQs needing protocol details without administrative delays. Traditionally, master CDAs were established between the site and the Sponsor. Moving forward, I-ACT for Children will implement Master Site Agreements with site, sponsor and network, aiming to transcend the Master CDAs in upcoming collaborations. Within the European c4c consortium, a standardized CDA or cascading CDA has also been developed but due to grant constraints, meaning developed material that was being tested in the proof-of-viability separate studies within c4c could not be used within national network separate partnerships. Both networks work with a single point of contact at site level, also known as a site champion, who serves as the main contact person and advocates for trial facilitation through research networks. The site champion also keeps the national network hub updated on the site's progress and any challenges encountered.

In the EU, clinical trial sites are often categorized into university hospitals and regional hospitals, with considerable variation in size among them. It's crucial for sponsors to clearly communicate the nature and classification of these sites to facilitate successful trial management. However, this distinction is not always indicative of simplicity in contracting processes. In the US, although many university hospitals own smaller peripheral centers, leading to an assumption of streamlined contracting, yet the arrangement can complicate the process. When smaller centers have the autonomy to operate independently, they often achieve much faster contracting times. The primary advantage of such a system lies in the extensive referral network it provides, rather than simplification of contractual agreements.

### 3.2 Metrics of feasibility assessment

Five pediatric clinical trial studies were supported through this partnership, encompassing neurology (2/5), cardiology (1/5), infectiology (1/5), and dermatology (1/5) therapeutic areas. All trials involved rare diseases, defined as affecting less than 1 in 2,000 people, and included early outreach and engagement activities, with two trials featuring trial facilitation and summary of site advantages, difficulties and network recommendation, and one trial involving CDA and FQ completion.

The trials were conducted across 17 different European countries, involving a total of 202 hospital departments as trial sites. If not accounting for multiple trials within the same country, the total count would be 32 countries. The first phase of site identification, the early engagement questionnaire, pre-feasibility or EES, had a response rate of 70% of 142 sites. The completion of the EES questionnaire for the five trials had a median of 38 days (IQR 20 days). A quality check within RedCap was completed for all site responses, and corrections were principal investigator (PI) experience reported, inaccuracies of site capabilities based on previous experience working with the department/hospital site, as well as recruitment capabilities. For the first trial, the CDA and FQ process was conducted, with a completion within 54 days or roughly 2 months for 7 countries. One of the main delaying factors was the CDA completion, varying between sites from 1 day to 30 days.

Geographical area and activity metrics are included in Table 2. Case-examples of trial facilitation per activity are summarized in Table 3. The most frequently requested activities include Early Engagement Survey (EES) or pre-feasibility, CDA and Trial Facilitation. The EES phase brought its own set of challenges, as demonstrated in Trials 2 through 5. For instance, Trial 2 identified the need for direct, language-specific peer support to address issues like unresponsiveness. The influence of external factors, such as pandemic-induced regulatory adjustments, on Trials 3 and 4, necessitated innovative solutions like a “wave-model” for trial outreach and reorganization of the clinical trial unit. The “wave-model” approach implies that countries were not approached simultaneously to participate in the trials; instead, they were contacted in successive “waves” of prioritization based on each country's site response rate within a given wave. The qualitative analysis also revealed that some sites, as noted in Trials 3–5, initially declined participation due to various misunderstandings or constraints. However, leveraging personal connections resulted in a significant recovery, with a 28% re-inclusion rate for sites that had previously declined.

Moreover, for Trials 1–5 during the EES and FQ stages, it was recognized that over half of the completed questionnaires needed adjustments due to misinterpretations by the Principal Investigators (PI) or site team. Comparing similarly capable sites across countries highlighted the necessity for such adaptations, predominantly in areas like site availabilities and recruitment estimates. Comprehensive summary reports were then crafted, emphasizing the pros and cons, strategic recommendations, and evaluations relative to the sponsor's goals.

In addition to these specific cases, a qualitative analysis was undertaken, examining internal communications. This deep dive aimed to pinpoint the key distinctions and hurdles faced across various continents, thereby enriching the overall understanding and approach to future trial facilitations.

For two trials, a summary of the early engagement as well as the FQ analysis was necessary to give the sponsor an overview of specifities per site, foreseen challenges for site opening and the networks recommendation for inclusion. The experienced based time collection required 50 min per summary. The summary included benefits of the site, potential hurdles and suggestions for control during the visit, as well as a concluding recommendation to the trial team. The sponsor discussions confirmed the usefulness of having the site's information summarized in one or two pages, instead of the 10–24 pages included within the questionnaires.

## 4 Discussion

In the last four years, pediatric clinical research networks have partnered across the Atlantic to improve the site identification quality and efficiency with early promising metrics. Trials were conducted across 17 European countries with 202 participating hospital sites. The initial phase, EES, had a 70% response rate from 142 sites, and sites took a median of 38 days (IQR 20 days) to complete the questionnaire for five trials. The trial support activities, including EES, CDA and feasibility questionnaires, could be completed across 7 countries within 84 days. Factors such as precontracted sites, limited scope of studies, changes in timelines, COVID-related disruptions, and a learning curve for collaboration need to be considered when evaluating these metrics. Sponsors have confirmed additional benefits of transatlantic clinical trial facilitation, aside from the promising efficiency of site identification. These include the quality control and summarization of questionnaire findings, establishing personal connections with sites through peer-to-peer communication to increase willingness and understanding to participate in often lengthy questionnaires without reimbursement. Moreover, having a standby facilitator during the trial start-up has been requested often due to the high prevalence of unforeseen hurdles and communication difficulties.

The promising metrics are partly supported by the peer-to-peer connection networks have to the sites and the real-time evaluation of the capability and interest to participate per trial type. The availability standardized CDA processes is highly valued by sponsors, which is incorporated within the US network and was developed during the c4c project. The use of standardized or cascading CDA's in a European setting is available via through the c4c *Stichting* after grant completion (https://conect4children.eu/). Comparison of early-stage pharmacological clinical trials facilitation by networks is largely lacking in literature. The performative metrics of the c4c trials by Degraeuwe et al. (2023) showed feasibility completion within 30 days in 2 high-performative research networks in Belgium and the Netherlands, showing the value of expertise in the pediatric landscape as well as the benefit of standardized contracting (18). Moreover, *Tuft CSDD* reported site identification requiring at average 8 weeks including both adult and pediatric studies, 50% higher than our findings. This metric does not account for national adaptations, quality control or summary report services.

Furthermore, during the pre-feasibility phase, the networks’ expertise in the respective pediatric landscape allowed for recommendations to be made to increase country outreach selection and inclusion of quality sites. Responses were corrected and compared between sites and countries. For example, leveraging personal connections resulted in a significant recovery, with a 28% re-inclusion rate for sites that had previously declined. This resulted in increased quality of site and country for trial inclusion, including a theoretical higher recruitment of the respective studies. An important best practice for all in clinical trial facilitation is the implementation of a handover process, as sites may be contacted by multiple staff members from the sponsor or clinical research organization (CRO), which can cause confusion and reluctance to participate. Utilizing a trusted and familiar member of the research network to initiate contact with the site can prioritize the interests of both the site and sponsor/, and a smooth handover to the sponsor's/CRO's trial manager can establish a strong foundation for future communication and conduct of the clinical trial. In addition to these successful case-analysis in Europe through collaboration, c4c has developed internal standardized processes to address trial support across the trial lifecycle with multiple sponsors and across indications, which all pediatric clinical research networks have built on, implemented, and benefited from. I-ACT for Children has also established best practices that are exemplary for other countries and continents. These include the implementation of a master-CDA and centralization of regulatory authorities.

Moreover, recruitment has been identified by the EMA as a specific challenge in pediatric clinical trials (19). Currently, retention across the studies showed an average 20% loss over time and most clinical trial sponsors or CRO do not use the pre-existing networks or actively standardize and maintain the infrastructure. A call-to-action from regulators to use pre-existing established networks could be beneficial in aligning interest across and collaborations with multiple stakeholders. These networks can include overarching networks, as mentioned, or discipline specific networks such as the European Cystic Fibrosis Clinical Trial Network (ECFS-CTN; www.ecfs.eu/ctn) (20). The recruitment challenge will become more apparent when facing upcoming rare disease trials requiring scattered recruitment within pediatric and adult cases globally.

Furthermore, there is a growing demand for versatile networks that extend beyond traditional domains of pediatric clinical trials, including areas such as psychology, dermatology, and surgery. Additionally, there is a need for a wider range of trial types aside from drugs initially marketed for adults. The demand for conducting clinical trials involving ultra-rare diseases, vaccines, medical devices, drug repurposing, and academic-driven (intent-to-label) studies in children is increasing. National networks expertise can be beneficial to guide sponsors to select relevant and realistic drug trial types as well as improve racial and ethnic representation in pediatric clinical trials (21, 22). There is a continuing necessary investment into other areas of the world (23–25).

*Greenberg* et al. *(*2022) has noted that pediatric clinical research networks have been constructed similarly and with internal standardization, and should be globally interoperable in 5-years (13). To achieve global interoperability in pediatric clinical trials, there are several essential factors to consider. These include the widespread dissemination of network conduct and metrics, increased collaboration across continents, standardization of facilitation conduct and multi-stakeholder financial support. To prepare for the upcoming wave of pediatric clinical trials, it is also beneficial to include/promote additional national networks, such as those in Japan.

Limitations of this study include the timeframe of 3-years including 5 clinical trials, limiting the calibration of the metric results to other networks and/or reaching a higher learning curve of trial facilitation through networks by multistakeholder. As most of the trials are still ongoing, there is a lack of data available regarding recruitment and dropout rates during the later stages of the trials. Moreover, consultation of the sponsors included in the case study has been indirectly incorporated in Table 3 yet not directly documented. Furthermore, it was not possible to conduct a post-hoc analysis of recruitment and dropout rates at individual sites. Three out of five trials were conducted as rescue studies through research networks, which limited the ability to showcase success ratios of site selection and start-up timelines. It is worth noting that all the included trials originated from US-based pharmaceutical companies, and no service requests were received from a European-based setting.

We propose the utilization of the network directly to maximize time and budget efficiency in pediatric clinical trials. Implementing rescue strategies, which involve additional interventions to salvage trials, not only increase costs and burdens but also lead to frustration among the principal investigator (PI) and trial sites. These rescue strategies often result in repeated and consecutive requests for FQ assessments of the same protocol by various stakeholders, including pharmaceutical companies, CROs, and the network itself. Such repetitive requests negatively impact the quality of data delivered and can disrupt the smooth operation of clinical trial units.

## 5 Conclusion

Partnership between European collaborative national pediatric clinical research networks and the US-network I-ACT for Children has supported site identification of global pediatric clinical trials. This illustrates one method for the importance of early engagement with sponsors, promoting early metrics and implementation of effective communication systems.

## Data Availability

The raw data supporting the conclusions of this article will be made available by the authors, without undue reservation.
